# Generation of potentially inhibitory autoantibodies to ADAMTS13 in coronavirus disease 2019

**DOI:** 10.1038/s41598-023-37405-5

**Published:** 2023-06-28

**Authors:** Adrian A. N. Doevelaar, Martin Bachmann, Bodo Hölzer, Felix S. Seibert, Benjamin J. Rohn, Panagiota Zgoura, Oliver Witzke, Ulf Dittmer, Thorsten Brenner, Krystallenia Paniskaki, Serap Yilmaz, Rita Dittmer, Sonja Schneppenheim, Jochen Wilhelm, Ulrik Stervbo, Nina Babel, Ulrich Budde, Timm H. Westhoff

**Affiliations:** 1 Medical Department 1, University Hospital Marien Hospital Herne, Ruhr-University Bochum, Hölkeskampring 40, 44625 Herne, Germany; 2 Department of Intensive Care and Ventilatory Medicine, Asklepios Klinikum Hamburg Harburg, Hamburg, Germany; 3 Department of Infectiology, University Hospital Essen, University of Duisburg-Essen, Essen, Germany; 4 Institute for Virology, University Hospital Essen, University of Duisburg-Essen, Essen, Germany; 5 Department of Anesthesiology and Intensive Care Medicine, University Hospital Essen, University of Duisburg-Essen, Essen, Germany; 6 Center for Translational Medicine, University Hospital Marien Hospital Herne, Ruhr-University Bochum, Herne, Germany; 7 Department of Hemostaseology, MEDILYS Laborgesellschaft mbH, Hamburg, Germany

**Keywords:** Immunological disorders, Inflammatory diseases, Thrombocytopaenic purpura, Infection

## Abstract

It has recently been shown that von Willebrand factor (VWF) multimers contribute to immunothrombosis in Coronavirus disease 2019 (COVID-19). Since COVID-19 is associated with an increased risk of autoreactivity, the present study investigates, whether the generation of autoantibodies to ADAMTS13 contributes to this finding. In this observational prospective controlled multicenter study blood samples and clinical data of patients hospitalized for COVID-19 were collected from April to November 2020. The study included 156 individuals with 90 patients having confirmed COVID-19 of mild to critical severity. 30 healthy individuals and 36 critically ill ICU patients without COVID-19 served as controls. ADAMTS13 antibodies occurred in 31 (34.4%) COVID-19 patients. Antibodies occurred more often in critically ill COVID-19 patients (55.9%) than non-COVID-19 ICU patients and healthy controls (5.6% and 6.7%; p < 0.001), respectively. Generation of ADAMTS13 antibodies in COVID-19 was associated with lower ADAMTS13 activity (56.5%, interquartile range (IQR) 21.25 vs. 71.5%, IQR 24.25, p = 0.0041), increased disease severity (severe or critical in 90% vs. 62.3%, p = 0.019), and a trend to higher mortality (35.5% vs. 18.6%, p = 0.077). Median time to antibody development was 11 days after first positive SARS-CoV-2-PCR specimen. Gel analysis of VWF multimers resembled the constellation in patients with TTP. The present study demonstrates for the first time, that generation of ADAMTS13 antibodies is frequent in COVID-19, associated with lower ADAMTS13 activity and increased risk of an adverse disease course. These findings provide a rationale to include ADAMTS13 antibodies in the diagnostic workup of SARS-CoV-2 infections.

## Introduction

Coronavirus disease 2019 (COVID-19) is associated with micro- and macrovascular thrombotic events—a phenomenon, which has recently been described as “immunothrombosis”. Macrovascular events comprise both venous thrombembolism and arterial thrombotic events including myocardial infarction, stroke, and limb ischemia^1^. Microvascular thrombosis has preferentially been described by autopsy studies in the lungs and contributes to SARS-CoV-associated acute respiratory distress syndrome^2^.

We and others observed thrombotic microangiopathy (TMA) in one or more patients^3–5^. Recently, we demonstrated that COVID-19 is associated with a substantial increase in von Willebrand factor (VWF) concentrations, which can exceed the ADAMTS13 processing capacity resulting in the formation of large VWF multimers identical to thrombotic thrombocytopenic purpura (TTP)^5,6^. The ADAMTS13/VWF Antigen (VWF:Ag) ratio was thereby an independent predictor of severity of disease and mortality. In the present study we investigated whether the generation of antibodies to ADAMTS13 might contribute to this observation.

## Materials and methods

We performed an observational prospective multicenter study and enrolled 156 participants including 90 patients, who were hospitalized for COVID-19. All participants of the study had not been vaccinated against SARS-CoV-2 priorly. Patients were recruited at Ruhr-University Bochum (59 patients), University of Duisburg-Essen (2 patients), and Asklepios Klinikum Hamburg Harburg (29 patients), Germany. Assuming that the incidence of autoantibodies is 30% in COVID-19 patients and 5% in critically ill non-COVID-19 controls with an alpha-significance level of 0.05 and a power of 80%, 35 patients per group were required. Severity of COVID-19 disease was categorized into mild, meaning asymptomatic disease, moderate, defined as symptomatic disease without respiratory failure, severe, defined as respiratory failure without the need of mechanical ventilation, to critical, defined as respiratory failure with the need of mechanical ventilation or use of vasoactive agents. These categories were adopted from the guidelines of the Robert Koch Institute (RKI), Germany^7^. 30 healthy subjects and 36 non-COVID-19 patients, matching the criterions for critical disease severity from the RKI from the intensive care unit (ICU) served as controls. Last mentioned patients mainly suffered from pneumonia, sepsis or myocardial infarction. Demographic, clinical and hemostaseologic characteristics of patients at initial sample obtainment are summarized in Table 1.

**Table 1:** Baseline epidemiological, clinical and haemostaseological characterization of the study population.

	Healthy controls (n=30)	Critically ill patients without COVID-19 (n=36)	COVID-19 population (n=90)	COVID-19 with antibodies to ADAMTS13 (n=20)	COVID-19 without antibodies to ADAMTS13 (n=70)	P
Age (years)	33.5 (23.0)	70 (25.0)	66.5 (22.25)	67.5±14.83	64.13±14.77	0.37
Female	22 (73.3%)	16 (44.4%)	42 (46.7%)	9 (21.4%)	33 (78.6%)	0.8655
Male	8 (26.7%)	20 (53.6%)	48 (53.3%)	11 (22.9%)	37 (77.1%)	
Race						
White	30 (100%)	36 (100%)	89 (98.9%)	20 (22.7%)	69 (77.3%)	0.591
Asian	0 (0%)	0 (0%)	1 (1.1%)	0 (0%)	1 (100%)	
Disease severity						
Mild or moderate			29 (31.5%)	2 (10%)	27 (38.6%)	**0.0159**
Severe or critical			61 (68.5%)	18 (90%)	43 (61.4%)	
Outcome						
Alive			68 (75.6%)	13 (65.0%)	55 (78.6%)	0.2129
Dead			22 (24.4%)	7 (35.0%)	15 (21.4%)	
Serum creatinine concentration (mg/dL)			1.1 (1.0)	1.1 (0.925)	1.1 (1.275)	0.7999
Glomerular filtration rate (ml/min, MDRD)			>60	57.5 (46.25)	65.0 (60)	0.7284
C-reactive protein (mg/dL)			8.10 (11.86)	6.6 (10.275)	8.945 (12.722)	0.9904
Lactate dehydrogenase (U/L)			331 (220)	342 (257)	330 (215.5)	0.5506
White blood cell count (/nL)			7.1 (4.8)	10 (6.8)	7 (4.85)	0.1192
Platelet count (/nL)			242±109.825	250.5 (228.2)	219 (135.8)	0.4983
Hemoglobin (g/dL)			11.6±1.792	11.10±2.175	11.76±1.656	0.1461
D-dimer (mg/L)			0.977 (1.5152)	2.080 (2.0475)	0.92 (1.452)	0.2258
Fibrinogen (mg/dL)			6.995 (0.872)	6.18 (1.19)	7.0 (0.75)	0.1018
Activated partial thromboplastin time (s)			32.75 (12.05)	31.5 (25.3)	33.0 (9.5)	0.6315
International normalized ratio			1.18 (0.24)	1.21 (0.27)	1.17 (0.23)	0.1801
VWF:Ag (IU/mL)	97 (61,55)	239 (217)	326 (167)	380.5 (185.5)	310 (113)	0.1036
ADAMTS13 activity (%)	75.0 (22.5)	47.5 (28)	67.5 (28.5)	56.5 (21.25)	71.5 (24.25)	**0.0041**
ADAMTS13/VWF:Ag	82.04±30.71	28.20±22.69	20.23±9.427	12.6 (10.93)	21.6 (13.45)	**0.0125**
Antibodies to ADAMTS13 (%)	2 (6.7%)	2 (5.6%)	20 (22.2%)			
Concentration of ADAMTS13 antibodies	2.9 (3.65)	2.0 (4)		27 (13.5)	5 (5.25)	**<0.0001**

*COVID-19* Coronavirus Disease 2019, *MDRD* Modification of Diet in Renal Disease, *VWF:Ag* von Willebrand factor Antigen.

Data are presented as mean ± standard deviation for normally distributed parameters, otherwise in median and interquartile range.

P indicates comparison of COVID-19 patients with and without antibodies to ADAMTS13. P<0.05 was regarded significant (bold type).

ADAMTS13 activity and IgG-antibodies to ADAMTS13 were analyzed from citrate-plasma and serum using a chromogenic Technozym^®^ ADAMTS13 ELISA Kit and Technozym^®^ ADAMTS13 INH ELISA Kit (Technoclone, Vienna, Austria), respectively^8^. An ADAMTS13 antibody concentration of ≥16 U/mL was considered positive according to manufactures guidelines. VWF:Ag was measured using a sandwich ELISA with polyclonal antibodies^9^. The ADAMTS13/VWF:Ag ratio was calculated as (ADAMTS13 (IU/ml)/VWF:Ag (IU/ml) × 100). VWF multimer analysis was performed via sodium dodecyl sulfate agarose gel electrophoresis including a control sample containing normal VWF in each run to ensure proper conditions of the separation and blotting apparatus^10^. Parts of the study population and the control group have previously been described^11^. The study has been approved by the ethical committees of Ruhr-University Bochum (20-6886), University Hospital Essen (20-9214-BO) and the Medical Association Hamburg. Written informed consent was given by the participants before entry into the study and all experiments were performed in accordance with relevant guidelines and regulations.

## Results

### Initial sample data

**Figure 1:**
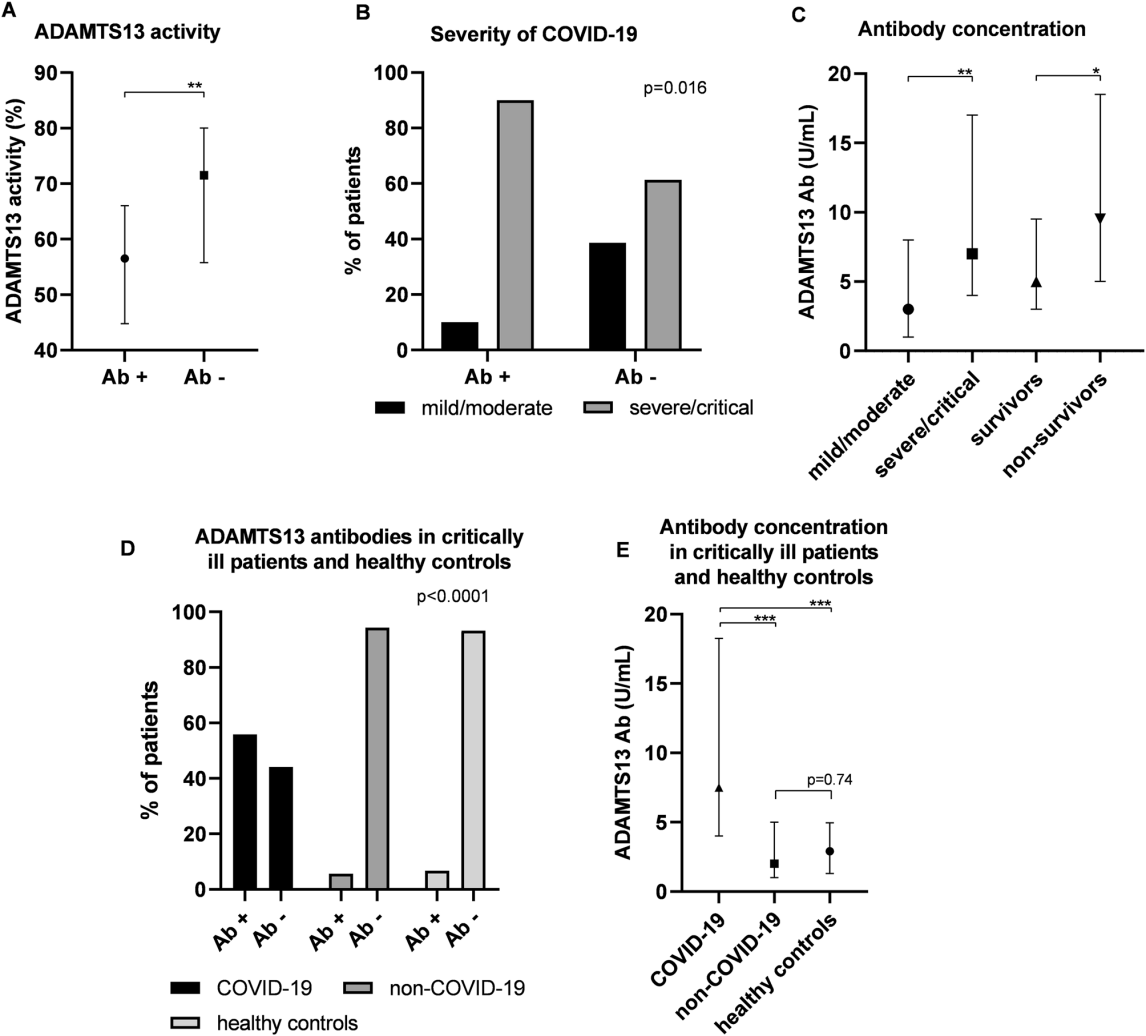
Baseline data of COVID-19 patients. (**A**) ADAMTS13 activity (%) in dependence on antibody (Ab) status. (**B**) Severity of disease in dependence on Ab status. (**C**) Ab concentration (U/mL) in dependence on disease severity and mortaliy. (**D**) % of critically ill COVID-19 and non-COVID-19 patients and healthy controls. (**E**) Ab concentration in critically ill COVID-19 vs. non-COVID-19 patients and healthy controls. Data are presented in % of patients with chi squared p-values, or median and interquartile range, respectively.

Median time to initial sample obtainment was 4 days after first positive PCR specimen, and 3 days after admission, respectively. Compared to healthy controls VWF:Ag (iU/mL) was significantly higher in patients with COVID-19 (326 iU/mL, interquartile range (IQR) 163 vs. 97 iU/mL, IQR 61, p<0.0001). Median ADAMTS13 activity was 67.5%, IQR 28.5, in COVID-19 patients vs. 75.5%, IQR 22.5, in healthy controls (p=0.017). The ADAMTS13/VWF:Ag ratio was substantially lower in COVID-19 patients (20.2±9.4 vs. 82.0±30.7, p<0.0001). More patients with COVID-19 already had elevated antibodies to ADAMTS13 at initial sample obtainment compared to healthy controls (22.2% vs. 6.7%, chi squared p=0.0565). Median concentration of ADAMTS13 antibodies at initial sample obtainment was 27 U/mL (IQR 13.5) and median ADAMTS13 activity was significantly lower in those patients with ADAMTS13 antibodies (56.5%, IQR 21.25 vs. 71.5%, IQR 24.25, p=0.0041, Fig. 1A). Spearman correlation analyses revealed a significant negative correlation between ADAMTS13 antibody concentration and ADAMTS13 activity (r=− 0.321, p=0.002). Hematological parameters did not significantly differ depending on ADAMTS13 antibody status. Severity of disease differed in dependence of antibody status at hospital admission. Patients with ADAMTS13 antibodies at baseline evaluation had a significantly higher disease burden compared to subjects without antibodies (chi squared p=0.016, Fig. 1B). Moreover, baseline ADAMTS13 antibody concentration differed significantly dependent on disease burden and outcome (Fig. 1C). In univariate binary logistic regression analyses baseline ADAMTS13 antibody concentration (regression coefficient (r)=− 0.04, p=0.046), ADAMTS13 activity (r=0.038, p=0.009), VWF:Ag (r=-0.006, p=0.001) and ADAMTS13/VWF:Ag ratio (r=0.114, p=0.001) had a significant impact on mortality.

### Subgroup analysis of critically ill patients

**Figure 2:**
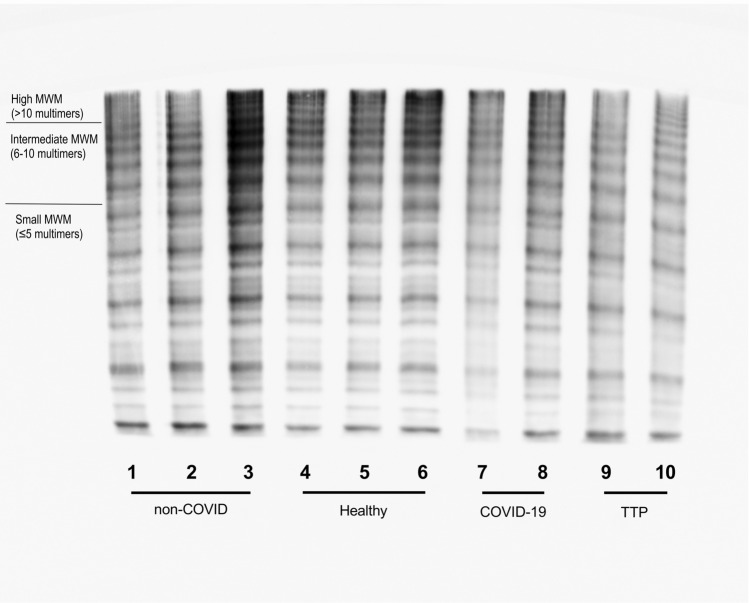
Von Willebrand factor multimers in a medium resolution gel (1.8% LGT-agarose, images taken from different gels) of (7-8) two patients with severe COVID-19, increased release of VWF and autoantibodies to ADAMTS13 and (1-3) three critically ill patients without COVID-19, (4 and 6) healthy controls, (5) pooled plasma of healthy controls, and (9-10) two patients with acute thrombotic thrombocytopenic purpura prior to initiation of treatment. The gel shows smear and a decrease of largest multimers predominantly in the patient lanes of both COVID-19 patients and those with thrombotic thrombocytopenic purpura.

The control group of critically ill patients without COVID-19 was comparable to the COVID-19 population regarding age, gender, and cardiovascular comorbidities. Substantially more critically ill patients with COVID-19 had antibodies to ADAMTS13 compared to non-COVID-19 ICU patients (55.9% vs. 5.6%, OR 21.53, 95% CI 4.609–98.15, p < 0.0001, Fig. 1D). ADAMTS13 antibody status of critically ill non-COVID-19 patients did not differ from healthy controls (5.6% vs. 6.7%, p = 0.74). Median ADAMTS13 antibody concentration was substantially higher in critically ill COVID-19 patients (7.5 vs. 2 U/mL, p = 0.0002, Fig. 1E). Representative gel analyses of patients with COVID-19 having increased VWF concentrations and antibodies to ADAMTS13, critically ill patients without COVID-19 and, for reasons of comparison, patients with acute TTP are presented in Fig. 2 and Supplementary Fig. 1.

### Follow-up data

**Figure 3:**
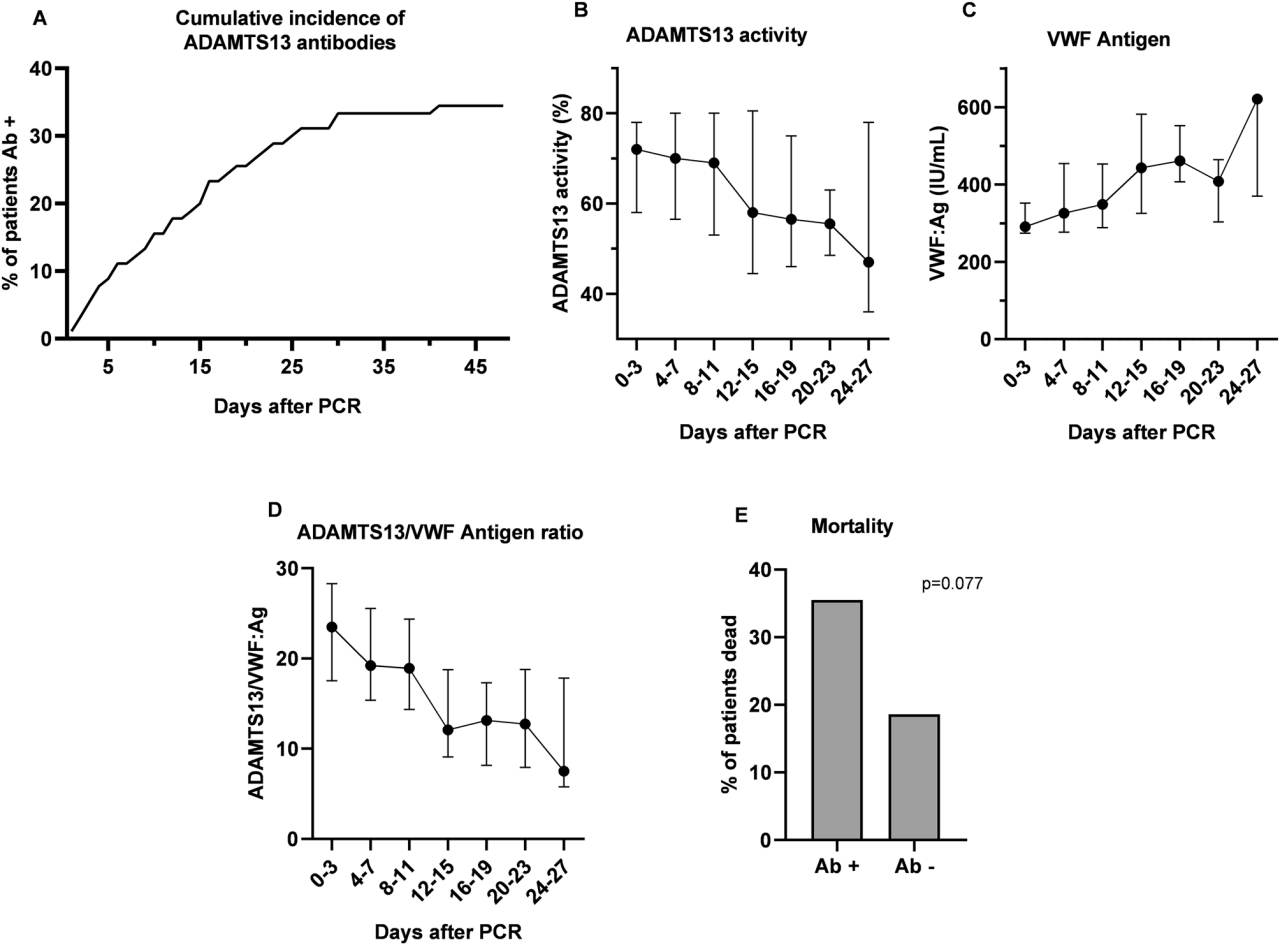
(**A**) cumulative incidence of ADAMTS13 antibodies (Ab), (**B**) ADAMTS13 activity, (**C**) von Willebrand factor Antigen (VWF:Ag), (**D**) ADAMTS13/VWF:Ag ratio, and (**E**) mortality in dependence on ADAMTS13 antibody status in the course of Coronavirus disease. Data are presented in median and interquartile range.

In 37 (41.1%) COVID-19 patients, follow-up blood samples were available. 11 (29.7%) of initially negative tested patients converted to positive ADAMTS13 antibody status during their hospital stay within a median time of 11 days after first positive PCR specimen and 9 days after hospital admission, respectively. Thus, among the whole COVID-19 study population, at least 31 patients (34.4%) developed ADAMTS13 antibodies (Fig. 3A). In follow-up samples median ADAMTS13 activity and ADAMTS13/VWF:Ag ratio decreased over time, with increasing VWF:Ag levels (Fig. 3B–D). Spearman correlation analyses revealed a significant negative correlation between ADAMTS13 antibody concentrations and ADAMTS13 activity in follow up samples (r = − 0.380, p<0.001). Furthermore, development of ADAMTS13 antibodies in the 11 initially negative tested patients were associated with significantly lower ADAMTS13 activity in paired t-test (mean of difference −20.18%, 95% CI −39.75 to −0.61, p = 0.0444), missing statistical significance in VWF:Ag levels, likely due to the small number of cases (mean of difference +43.43 U/mL, 95% CI −117.7 to +204.6, p = 0.5341). Overall, ADAMTS13 antibody positive patients had a worse outcome than antibody negative patiens (35.5% vs. 18.6%, chi squared p = 0.077, Fig. 3E).

## Discussion

In summary, the present findings show that (1) the higher VWF concentrations and the lower ADAMTS13, the higher the probability of a severe course of COVID-19 including risk of death. (2) The present study demonstrates for the first time, that generation of antibodies against ADAMTS13 is a frequent and unique finding in COVID-19 occurring in approximately one third of hospitalized patients and is associated with a lower ADAMTS13 activity suggesting an inhibitory effect on the protease. (3) The study shows that not only the presence but also the concentration of ADAMTS13 antibodies predicts the severity of COVID-19.

Lower ADAMTS13 activity and ADAMTS13/VWF:Ag ratio have been associated with an increase in morbidity and mortality before^6,11^. The present study adds the information that not only the massive release of VWF but also the generation of autoantibodies to ADAMTS13 contribute to the decrease of the ADAMTS13/VWF:Ag ratio. Autoantibodies to ADAMTS13 have rarely been investigated in the context of COVID-19 so far. In recent literature we found only two small case series with critically ill COVID-19 patients, which detected ADAMTS13 antibodies in only one out of 13 patients^12,13^.

In general, positive ADAMTS13 antibodies along with a markedly reduced ADAMTS13 activity are diagnostic criteria for TTP. Noteworthy, none of the patients in our study population developed severe thrombopenia <50,000/µl. Thus, the potentially inhibitory effect is weaker than in TTP. Moreover, it has to be kept in mind that low concentrations of apathogenic ADAMTS13-antibodies may occur in healthy persons as well. For two reasons, however, it is unprobable that the present autoantibodies were completely apathogenic: First, detection was associated with a lower ADAMTS13 activity suggesting an inhibitory effect on the protease. Second, both the presence and the concentration of ADAMTS13 antibodies predicted the severity of COVID-19. Since the presence and concentration of antibodies correlated with severity, the duration of COVID-19 may be a risk factor for development of ADAMTS13 antibodies. In our study an ELISA assay was used to detect ADAMTS13 antibodies, which does not directly provide information about functional aspects of these antibodies. Therefore, a Bethesda assay might be a reasonable addition in future studies.

The immune response to SARS-CoV-2 is associated with an increased risk of autoreactivity. Hence, antibodies to phospholipids and interferon have been described during the pandemic^14,15^. Moreover, lupus- and rheumatoid arthritis-like antibody patterns have been observed in COVID-19^16^. Accordingly, critically ill patients with COVID-19 display hallmarks of extrafollicular B cell activation and shared B cell repertoire features typical of autoimmune settings^17^. Interestingly, the proportion of patients developing an ANA titer of ≥1:160 in another study was very similar (35.6%)^16^ to the proportion of subjects developing ADAMTS13 antibodies in the present study (34.4%). SARS-CoV-2 thereby increases the risk of thrombotic microangiopathy by potentially two synergistic mechanisms: First, the ubiquitous endothelial damage induces an excessive release of VWF, exceeding the protease activity of physiological concentrations of ADAMTS13. Second, the SARS-CoV-2 induced autoreactive inflammatory milieu leads to the generation of autoantibodies to ADAMTS13, which potentially reduces ADAMTS13 activity and thereby may—in addition to the excess of VWF—constitute an adjunct to the generation of immunothrombosis. Both of these mechanisms may yield an increased risk of intravascular large and ultralarge VWF multimers with TMAs resembling TTP.

We provide representative gel analyses of patients with COVID-19 having increased VWF concentrations and antibodies to ADAMTS13, critically ill patients without COVID-19 and patients with acute TTP. COVID-19 patients resemble the gels of patients with TTP. In both entities ultralarge multimers accumulate in the microthrombi and therefore show reduced concentrations in the circulation and corresponding gel analyses during the acute phase of disease.

What are the therapeutic consequences of this study? Plasma exchange constitutes a therapeutic option, which would be able to reduce both the excessive VWF and the antibodies to ADAMTS13 and—moreover—would deliver ADAMTS13. It could thereby reestablish the physiological balance between VWF and its protease. In first case series plasma exchange was used to attenuate circulating cytokines and inflammatory mediators in critically ill patients with COVID-19. Case series from Barcelona and Heidelberg describe favorable effects on parameters of inflammation and clinical outcome^18,19^. In a cohort in Oman, 11 critically ill patients underwent plasma exchange, which was associated with higher extubation rates and lower mortality^20^. We performed plasma exchange in 25 critically ill COVID-19 patients with acute respiratory distress syndrome, leading to a significant decrease in VWF:Ag and increased ADAMTS13 activity^21^. Our findings provide a rationale beyond the elimination of cytokines to suggest plasma exchange as a therapeutic strategy in COVID-19. A limitation of the study lies in its cross-sectional character. A larger cohort of patients followed longitudinally could further underline the significance of our findings.

## Conclusions

In conclusion, the present study demonstrates for the first time, that a substantial part of patients with COVID-19 develop autoantibodies to ADAMTS13 in the course of their disease, occurring in approximately one third of hospitalized patients, which could not be seen in ICU patients without COVID-19. Occurrence of antibodies is associated with a significantly lower ADAMTS13 activity, which causes a decreased degeneration of large and ultralarge vWf multimers likely contributing to SARS-CoV-2 induced immunothrombosis. The impact of each individual parameter on morbidity and mortality of COVID-19 is consistent with the physiology of this primary hemostasis system and very much in line with the pathophysiology of thrombotic microangiopathy: The excess of VWF, the decrease of ADAMTS13, the occurrence and the concentration of antibodies to ADAMTS13: Each of these individual parameters independently predicts adverse outcome. The findings that the antibodies predict outcome in a dose-dependent manner and that they are associated with impaired ADAMTS13 activity suggest a potential functional relevance. These findings provide a rationale to consider plasma exchange as a therapeutic option in COVID-19 and to include VWF, ADAMTS13 activity, and antibodies to ADAMTS13 in the diagnostic workup. Functional aspects of ADAMTS13 antibodies using a Bethesda assay should be subject of future studies.

## Supplementary Information

Supplementary Figure 1.

## Data Availability

The datasets used and/or analysed during the current study are available from the corresponding author on reasonable request.

## Abbreviations

VWF: von Willebrand factor
COVID-19: Coronavirus Disease 2019
TMA: Thrombotic microangiopathy
VWF:Ag: VWF Antigen
IQR: Interquartile range
RKI: Robert Koch Institut
ICU: Intensive care unit
TTP: Thrombotic thrombocytopenic purpura

## References

[1] Klok FA (2020). Incidence of thrombotic complications in critically ill ICU patients with COVID-19. Thromb. Res..

[2] Connors JM, Levy JH (2020). Thromboinflammation and the hypercoagulability of COVID-19. J. Thromb. Haemost..

[3] Jhaveri KD (2020). Thrombotic microangiopathy in a patient with COVID-19. Kidney Int..

[4] Airoldi A (2020). COVID-19-related thrombotic microangiopathy in a cirrhotic patient. Dig. Liver Dis..

[5] Doevelaar AAN (2021). von Willebrand Factor Multimer Formation Contributes to Immunothrombosis in Coronavirus Disease 2019. Crit. Care Med..

[6] Mancini I (2021). The ADAMTS13-von Willebrand factor axis in COVID-19 patients. J. Thromb. Haemost..

[7] STAKOP. (ed Robert Koch Institut) 6 (2022).

[8] Miyata T, Kokame K, Banno F (2005). Measurement of ADAMTS13 activity and inhibitors. Curr. Opin. Hematol..

[9] Cejka J (1982). Enzyme immunoassay for factor VIII-related antigen. Clin. Chem..

[10] Budde U (2008). Detailed von Willebrand factor multimer analysis in patients with von Willebrand disease in the European study, molecular and clinical markers for the diagnosis and management of type 1 von Willebrand disease (MCMDM-1VWD). J. Thromb. Haemost..

[11] Doevelaar AAN (2021). von Willebrand factor multimer formation contributes to immunothrombosis in coronavirus disease 2019. Crit. Care Med..

[12] Nazy I (2021). Platelet-activating immune complexes identified in critically ill COVID-19 patients suspected of heparin-induced thrombocytopenia. J. Thromb. Haemost..

[13] Alharthy A, Faqihi F, Balhamar A, Memish ZA, Karakitsos D (2020). Life-threatening COVID-19 presenting as stroke with antiphospholipid antibodies and low ADAMTS-13 activity, and the role of therapeutic plasma exchange: A case series. SAGE Open Med. Case Rep..

[14] Zuo Y (2020). Prothrombotic autoantibodies in serum from patients hospitalized with COVID-19. Sci. Transl. Med..

[15] Bastard P (2020). Autoantibodies against type I IFNs in patients with life-threatening COVID-19. Science.

[16] Woodruff MC, Ramonell RP, Lee FE, Sanz I (2020). Broadly-targeted autoreactivity is common in severe SARS-CoV-2 Infection. medRxiv.

[17] Woodruff MC (2020). Extrafollicular B cell responses correlate with neutralizing antibodies and morbidity in COVID-19. Nat. Immunol..

[18] Fernandez J (2020). Plasma exchange: An effective rescue therapy in critically Ill patients with coronavirus disease 2019 infection. Crit. Care Med..

[19] Morath C (2020). Plasma exchange in critically ill COVID-19 patients. Crit. Care.

[20] Khamis F (2020). Therapeutic plasma exchange in adults with severe COVID-19 infection. Int. J. Infect. Dis..

[21] Seibert FS (2022). Effect of plasma exchange on COVID-19 associated excess of von Willebrand factor and inflammation in critically ill patients. Sci. Rep..

