# Supplementary figures and images for: Generation of potentially inhibitory autoantibodies to ADAMTS13 in coronavirus disease 2019
Source: Sci Rep. 2023 Jun 28;13:10501. doi: 10.1038/s41598-023-37405-5 (PMC10307883; doi:10.1038/s41598-023-37405-5)

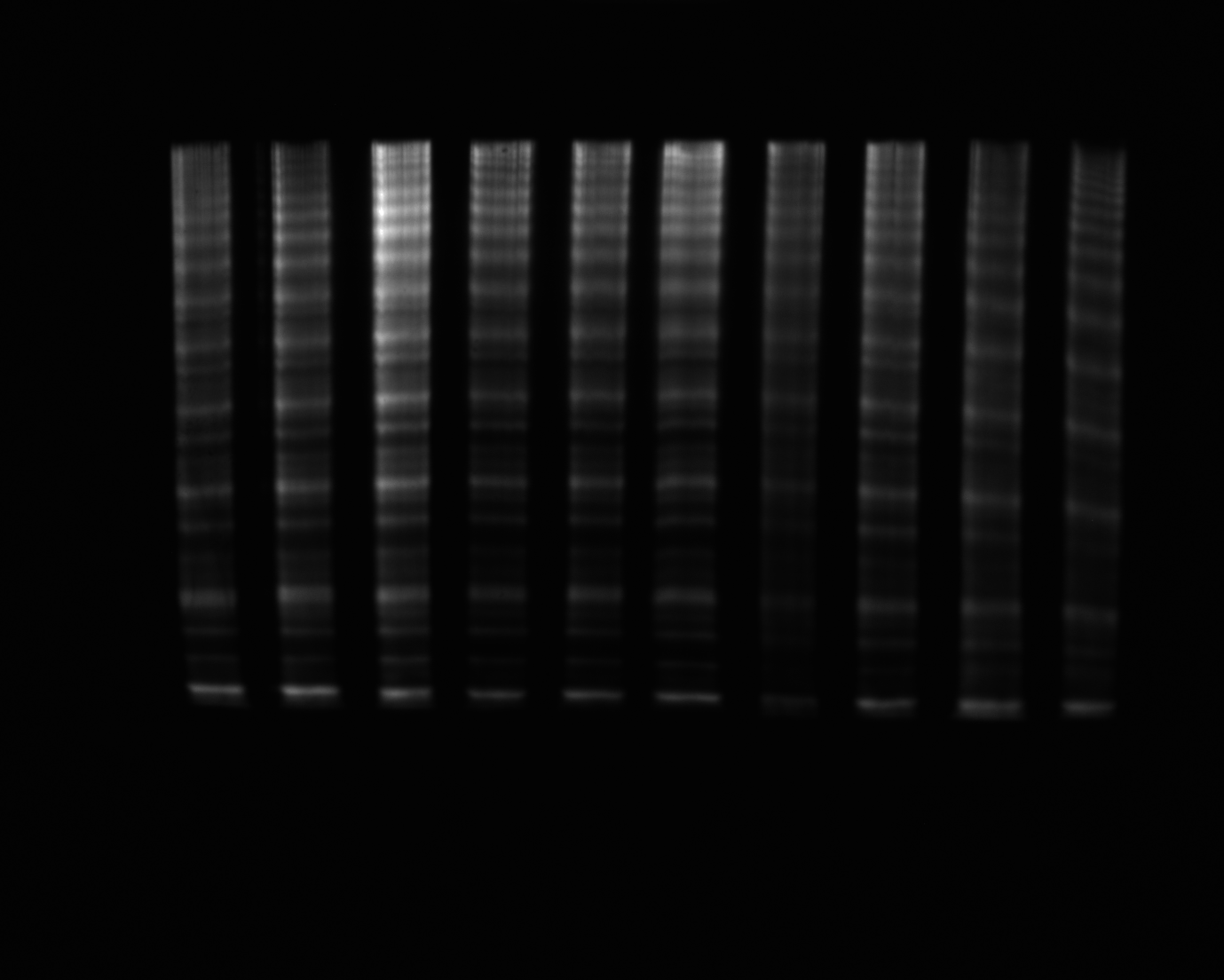

Supplement: Supplementary file 1 — Supplementary Figure 1. [file 41598_2023_37405_MOESM1_ESM.tif]
